# High Resolution HLA-A, HLA-B, and HLA-C Allele Frequencies in Romanian Hematopoietic Stem Cell Donors

**DOI:** 10.3390/ijms25168837

**Published:** 2024-08-14

**Authors:** Andreea Mirela Caragea, Radu-Ioan Ursu, Ion Maruntelu, Maria Tizu, Alexandra-Elena Constantinescu, Adriana Tălăngescu, Ileana Constantinescu

**Affiliations:** 1 Department of Immunology and Transplantation Immunology, “Carol Davila” University of Medicine and Pharmacy, 022328 Bucharest, Romania; andreea.m.caragea@gmail.com (A.M.C.); ion.maruntelu@drd.umfcd.ro (I.M.); maria.tizu@drd.umfcd.ro (M.T.); alexandra-elena.constantinescu0720@stud.umfcd.ro (A.-E.C.); adriana.oprea@drd.umfcd.ro (A.T.); ileana.constantinescu@imunogenetica.ro (I.C.); 2 Center for Immunogenetics and Virology, Fundeni Clinical Institute, 022328 Bucharest, Romania; 3 Department of Medical Genetics, “Carol Davila” University of Medicine and Pharmacy, 020021 Bucharest, Romania

**Keywords:** HLA-A, HLA-B, HLA-C, allele frequencies, transplantation immunogenetics

## Abstract

The HLA genes are associated with various autoimmune pathologies, with the control of the immune response also being significant in organs and cells transplantation. The aim of the study is to identify the HLA-A, HLA-B, and HLA-C alleles frequencies in the analyzed Romanian cohort. We performed HLA typing using next-generation sequencing (NGS) in a Romanian cohort to estimate class I HLA allele frequencies up to a six-digit resolution. A total of 420 voluntary donors from the National Registry of Voluntary Hematopoietic Stem Cell Donors (RNDVCSH) were included in the study for HLA genotyping. Peripheral blood samples were taken and brought to the Fundeni Clinical Institute during 2020–2021. HLA genotyping was performed using the Immucor Mia Fora NGS MFlex kit. A total of 109 different alleles were detected in 420 analyzed samples, out of which 31 were for HLA-A, 49 for HLA-B, and 29 for HLA-C. The most frequent HLA-A alleles were HLA-A*02:01:01 (26.11%), HLA-A*01:01:01 (12.5%), HLA-A*24:02:01 (11.67%), HLA-A*03:01:01 (9.72%), HLA-A*11:01:01, and HLA-A*32:01:01 (each with 8.6%). For the HLA-B locus, the most frequent allele was HLA-B*18:01:01 (11.25%), followed by HLA-B*51:01:01 (10.83%) and HLA-B*08:01:01 (7.78%). The most common HLA-C alleles were HLA-C*07:01:01 (17.36%), HLA-C*04:01:01 (13.47%), and HLA-C*12:03:01 (10.69%). Follow-up studies are ongoing for confirming the detected results.

## 1. Introduction

The major histocompatibility complex (MHC) has garnered significant scientific attention in recent decades due to multiple studies conducted on the HLA (human leukocyte antigen) genes, molecules that are part of the MHC. The HLA system is one of the most polymorphic genetic systems found in the human genome [1].

In anthropological research, as well as in the field of organ and stem cell transplantation, precise HLA allele identification is crucial [1,2,3,4,5]. Next-generation sequencing (NGS) HLA genotyping is required due to the increasing number of HLA alleles discovered through multiple studies [6,7]. Resolving allelic ambiguities and establishing updated allele frequencies are two benefits of using NGS for HLA typing [6,8,9,10,11]. These benefits will help with more accurate and comprehensive applications of HLA types in the fields of research and clinical medicine [11,12,13,14,15].

Technological developments in next-generation sequencing (NGS) have had a major impact on HLA typing recently [6,16,17,18,19]. Massive, parallel, high-resolution HLA typing is made possible by these novel techniques, which also manage the typical phase ambiguity of HLA alleles [6,20,21,22,23,24,25]. Numerous NGS-based HLA typing techniques have been developed, including whole exome or genome sequencing data-derived typing and amplicon-based HLA sequencing target enrichment of HLA genes.

Analyzing HLA allele frequencies in the Romanian population has been the subject of very few studies. Furthermore, because of the low-resolution or insufficient locus description in these studies, detailed HLA information was not presented. A precise and comprehensive method for HLA allele distribution is desperately needed for HLA typing.

While previous research has examined the distribution of HLA alleles in the Romanian population, the objective of this study was to use next-generation sequencing (NGS) for high-resolution HLA typing (three-field) and to ascertain the HLA-A, -B, and -C allele frequencies in the Romanian population.

The aim of the current study is to identify the frequencies of the various HLA-A, HLA-B, and HLA-C alleles for the analyzed Romanian cohort.

## 2. Results

### 2.1. Allele Frequencies

A total number of 109 different alleles were detected in the 420 analyzed samples, out of which 31 were for HLA-A, 49 for HLA-B, and 29 for HLA-C.

The most frequent alleles for the HLA-A locus were represented by A*02:01:01 (26.11%), A*01:01:01 (12.5%), A*24:02:01 (11.67%), A*03:01:01 (9.72%), A*11:01:01, and A*32:01:01 (each with 8.6%).

The HLA-B allele with the highest frequency was B*18:01:01 (11.25%), followed by HLA-B*51:01:01 (10.83%) and HLA-B*08:01:01 (7.78%).

At the HLA-C locus, the most common alleles were HLA-C*07:01:01 (17.36%), HLA-C*04:01:01 (13.47%), and HLA-C*12:03:01 (10.69%).

### 2.2. HLA-A Alleles

Genotyping results for the HLA-A alleles can be viewed in Table 1.

The most frequently detected alleles for the HLA-A locus were represented by the A*02:01:01 (26.11%), A*01:01:01 (12.5%), A*24:02:01 (11.7%), A*03:01:01 (9.72%), A*11:01:01, and A*32:01:01 alleles (each with 8.6%) (Table 1).

The top most frequent eight alleles count together for 80% of all alleles, each with frequencies of approximately 3% or higher.

Seven out of the top eight alleles showed frequencies of 5% or higher, totalizing more than three-quarters (approximately 77%) of all detected alleles.

Three alleles with frequencies higher than 10% were detected, accounting together for more than half (50.28%) of all counted alleles (362/720 alleles), while the fourth most frequent allele revealed a frequency close to 10% (9.72%) (approximately 10% of all alleles).

A total of 15 of the lower frequency remaining 23 HLA-A alleles were detected in less than 1% of the studied cohort, accounting for 5.73% of all counted alleles.

### 2.3. HLA-B Alleles

HLA-B genotyping results can be viewed in Table 2.

The results identified 49 different HLA-B alleles, with the most frequently detected allele being the B*18:01:01 variant (allele frequency 11.25%) (Table 2).

A total of 28 out of the 49 HLA-B alleles had frequencies higher than 1% (653/729 alleles, 90.7%), out of which 4 revealed frequencies above 5% (34.85% of all counted alleles) and just 2 had frequencies higher than 10% (22.08%) (Table 2).

The top 2 HLA-B alleles account together for a combined frequency of 22.08%, both being identified in approximately 11% of all analyzed patients (HLA-B*18:01:01—11.25%, HLA-B*51:01:01—10.83%) (Table 2).

Just two alleles had frequencies ranging between 5% and 10%, combining for approximately 13% (B*08:01:01—7.78%, B*35:01:01—5%).

A total number of 45 alleles (approximately 92% of all observed HLA-B alleles) had frequencies lower than 5%, summing up for a combined frequency of approximately 65%.

Out of these alleles, 21 were rare, with frequencies lower than 1%, combining for a total frequency of approximately 9% (67 out of the total of 720 alleles).

Five of the rare variants were observed in a single individual each (0.14%), while another six were detected in two persons each (0.28%) and three were detected in three individuals (0.42%).

### 2.4. HLA-C Alleles

The HLA-C alleles identified in our study can be viewed in Table 3.

A total number of 29 HLA-C variants were detected (Table 3).

For two HLA-C alleles (C*12:12 and C*15:72), the NGS results came in four digits, a possible cause being the NGS library for the respective variants not yet fully completed.

The most frequently identified HLA-C allele was HLA-C*07:01:01 (17.36%), followed by the HLA-C*04:01:01 allele (13.47%).

Eight HLA-C alleles revealed frequencies of 5% or higher (Table 3).

The most frequently identified 8 variants add up to 532 out of all the 720 detected HLA-C alleles, counting for a total of 75.25%. Out of these variants, three had frequencies higher than 10%, while the other five alleles were identified in between 5% and 10% of all identified HLA-C alleles (Table 3).

The 3 top HLA-C alleles with frequencies higher than 10% sum up to more than 41% of all the detected HLA-C variants, with all the other 26 alleles having a combined frequency of approximately 59% (Table 3).

Apart from these three variants with frequencies of more than 10%, the C*06:02:01 variant also presented a high allele frequency at 9.44%, combining for a total of approximately 51% with the previously mentioned top three alleles.

A total of 21 HLA-C variants had frequencies lower than 5%, totalizing 178 of all the 720 analyzed alleles, with a combined frequency of approximately 25%.

Out of these, 9 variants showed frequencies ranging between 1% and 5% (42.85%), while the other 12 were revealed to be rare variants (frequencies < 1%).

In total, these 12 rare HLA-C variants had a combined frequency of approximately 5.3% (38 of all the 720 tested alleles). Five of these variants were identified in one single individual each (0.13%), while the other rare alleles showed frequencies of 0.41–0.97%.

**Figure 1:**
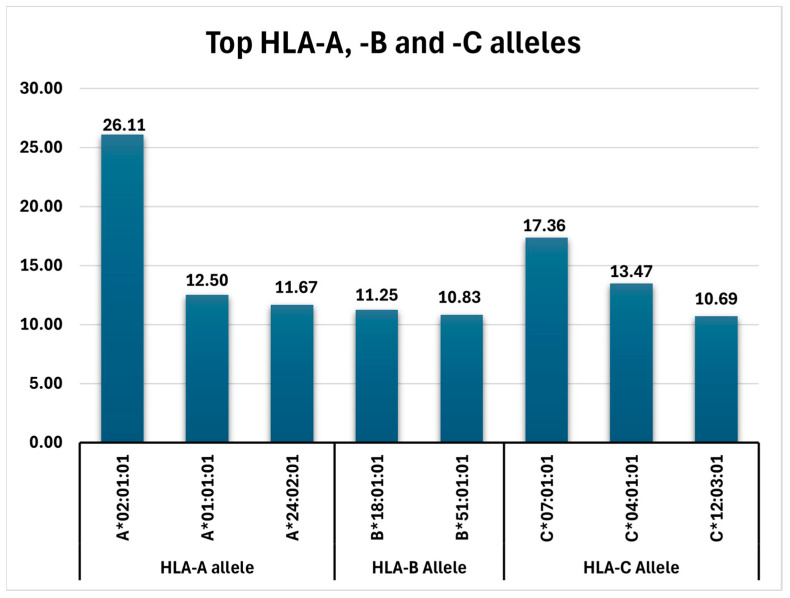
Top HLA-A, -B, and -C alleles identified in the analyzed Romanian cohort.

In concluding, the frequencies of all detected top alleles (HLA-A, HLA-B, and HLA-C variants with AF > 10%) can be viewed in Figure 1.

## 3. Discussion

The current study aimed to identify updated frequencies of HLA alleles at the level of six digits of resolution.

The obtained data can be used as additional information in the identification of cases in which the same four-digit or two-digit HLA types present different characteristics; this information is useful in studies involving the analysis of the HLA alleles associated with certain pathologies, with the analysis of adverse reactions related to medicines, immunological interaction studies, anthropological genetics, and in studies related to the Romanian population.

The immunogenetic profile of the studied Romanian cohort in terms of allele frequencies has been characterized [5]. Thus, the most frequent HLA alleles were the following:

- HLA-A: A*02:01:01 (26.11%), A*01:01:01 (12.5%), A*24:02:01 (11.7%), A*03:01:01 (9.72%), A*11:01:01, and A*32:01:01 (each with 6.25%);
- HLA-B: B*18:01:01 (11.25%), B*51:01:01 (10.83%), and B*08:01:01 (7.78%);
- HLA-C: C*07:01:01 (17.36%), C*04:01:01 (13.47%), and C*12:03:01 (10.69%).

### 3.1. HLA-A Alleles

As in the current research, the most frequent HLA-A allele in previously studied European populations was HLA-A*02 (also in past Romanian studies), with one exception, the Norway Sami population, where the most prevalent variant is HLA-A*03, the fourth most common in Romanians.

Even though, in most of the cases, the analysis was low resolution (France, England, Albania, Austria, Bosnia and Herzegovina, Croatia, Norway, Romania, Serbia, Slovakia, Sweden, and Switzerland), in countries with four-digit results, the analysis revealed the HLA-A*02:01 allele to be the most frequent (Belgium, Czech Republic, Finland, Germany, Greece, Italy, the Netherlands, Poland, Portugal, Kosovo, and Spain), while just one study (Bulgaria) was performed in high resolution, showing the same HLA-A allele as found in our Romanian cohort (HLA-A*02:01:01) [12,13,14,26].

The HLA-A*02:01:01 allele, the most common in our study group, is a highly prevalent variant in different worldwide Caucasian populations. The frequency identified in our cohort, of approximately 26%, is comparable to that observed in different global populations, such as South African Mixed Ancestry (AF 22%) and Caucasians (AF 26%), USA Caucasians (AF approximately 27%), certain Mexican populations (Chihuahua Tarahumara, Guadalajara Mestizo) (AFs of 22–25%), Caucasians from Brasil (AF 22%), and including European populations, such as the Spanish population from Canary Islands (AF 24%), Madeira, Portugal (AF approximately 25%), Polish (AF 28.5%), Bulgarians (AF 30%), English mixed ethnicity (AF 30%), or Russian populations (Vologda Region, Belgorod Region) (AFs 28% and 29%, respectively). The highest frequencies for this allele (AFs of around 40%) can be observed in indigenous populations from Northern and Central America, such as USA Arizona Gila River Pima (42.42%), Mexico Hidalgo Mezquital Valley/Otomi (40.28%), or USA New Mexico Canoncito Navajo (37.8%) [15].

The same comparable findings can be seen when analyzing the second most frequent allele in our cohort, HLA-A*01:01:01 (AF 12.5%), in this frequency range being found, again, were populations such as the Russian Belgorod and Vologda Regions populations (AFs of 9.5% and 10.5%), Brasilian Caucasians (AF approximately 10%), the Spanish Canary Islands population (AF 10.5%), and Polish or USA Caucasians (both with AFs of approximately 14%), but also other populations, such as Moroccan or South African Indian (AFs of 13% 14%) [15].

Similarities can also be observed with the third most common allele in our study group, HLAA*24:02:01 (AF 11.7%), found with comparable frequencies the same as the Russian Belgorod and Vologda Regions (AFs of 10.5% and 10%), Brasilian Caucasians (AF approximately 9%), the Spanish Canary Islands population (AF 9.3%), or Polish (9.2%) populations, but also in populations from Vietnam (13%), China (13.5–14%), India, or Nicaragua (both with approximately 14%) [15].

Multiple HLA-A alleles are associated with certain pathologies, with some being protective against and others causative for the disease. For example, among the HLA-A variants described in the literature in association with human disorders, the HLA-A*02:01 and HLA-A*24:02 alleles are correlated with an increased susceptibility for diabetes mellitus and insulin dependence [15,16,17,18,19,20,21,22,23,24,25,26,27]. In our study group, the frequencies of the HLA-A*02:01:01 and HLA-A*24:02:01 alleles are 26.11% and 11.67%, respectively, being two of the top three most frequent HLA-A alleles [15,16,17,18,19,20,21,22,23,24,25,26,27,28,29].

The HLA-A*03:01 allele is considered a risk factor for multiple sclerosis, playing an important role in the initiation phase of the disease [28,29], while the HLA-A*26:01 allele is associated with an increased predisposition for Behcet disease (BD) and the HLA-A*29:02 is involved in a higher susceptibility for autoimmune uveitis [28,29,30,31,32,33].

In our study group, the frequency of HLA-A*03:01:01 is one of the highest detected, close to 10% (9.72%), while the HLA-A*29:02:01 and HLA-A*26:01:01 variants are less frequent, being identified in 2.22% and 4.58%, respectively, of all analyzed individuals.

These findings, if confirmed population-wide through more and expanded research studies on Romanian cohorts, would indicate an approximately 10% population specific susceptibility for multiple sclerosis. This might represent a recommendation for screening programs which would need to include genetic testing for detecting the presence of the HLA-A*03:01:01 variant.

### 3.2. HLA-B Alleles

The HLA-B allele frequencies vary amongst the different European populations. The most prevalent alleles observed in previous studies were the HLA-B*07 variant (in France, Austria, Belgium, Finland, Germany, the Netherlands, Norway Sami population, Norway, Poland, Sweden, and Switzerland), the HLA-B*51 variant (Albania, Bulgaria, Greece, Italy, Portugal, and Kosovo), the HLA-B*44 allele (in England, Bosnian and Herzegovina, the Czech Republic, Slovakia, and Spain), and the HLA-B*35 variant (in Croatia and Serbia and a previous low-resolution HLA study on a Romanian cohort) [14].

In our analyzed Romanian population, the most frequent allele was HLA-B*18:01:01 (11.25%); almost equally common was the HLA-B*51:01:01 variant (10.83%).

Comparable high frequencies of the HLA-B*18:01:01 variant can be observed in certain populations which also revealed similar HLA-A allele frequencies to the current research, populations such as the Russian Belgorod and Novgorod Region (AF 12.7% and 6.8%), Polish (7.3%), or South African mixed ancestry (7%) populations [15].

Frequencies similar to the HLA-B*51:01:01 identified in our cohort have been described in populations such as Madeira Portugal (AF), USA Caucasians (approximately 9%), Russian Tundra Nentsi and Bashkortostan, Bashkirs (approximately 9% and 13%, respectively), and South African Caucasians (approximately 9), but even more in populations from Libya (10%), India (AFs 10–16%), China (11–15%), Canada (10%), Georgia (12%), or Saudi Arabia (14%) [15].

For the other frequent variants reported in the different European populations, the frequencies vary for Romanians, but that may be caused by the low-resolution results not being able to reveal the exact HLA allele.

For example, the most common HLA-B*44 detected allele in our study was B*44:02:01, being just the eighth most frequent (approximately 3%) of all HLA-B variants. If we calculate the HLA-B*44 combined frequency for all four detected alleles (B*44:02:01, B*44:03:01, B*44:05:01, and B*44:27:01), that would be approximately 9%. Similarly, the HLA-B*35 combined frequency for the detected variants (B*35:01:01, B*35:03:01, the fourth and fifth most common of all HLA-B alleles, B*35:02:01 and B*35:08:01) would be 12.36%, while the added HLA-B*51 allele frequency of the three detected alleles (B*51:01:01, B*51:05:01, and B*51:07:01) would be 11.53%.

In this context, the most frequent alleles in our Romanian cohort are HLA-B*35 (12.36%), HLA-B*51 (11.53%), and the HLA-B*18:01:01 allele (11.25%), followed by HLA-B*44 (approximately 9%). These results also confirm the outcomes of the previous Romanian low-resolution HLA study [12,13].

Even though the HLA-B*18:01:01 is the most frequent standalone allele, no other HLA-B*18 variants were detected.

The HLA-B*07 variant was much less common in Romania when compared to other European populations, with only two HLA-B*07 alleles being identified, but with average to low frequencies (B*07:02:01—3.75%, and B*07:05:01—0.56%).

Although the exact roles of certain HLA-B alleles (such as HLA-B*35:01:01, HLA-B*35:03:01, HLA-B*38:01:01, and HLA-B*14:02:01, a.o.) is yet to be fully understood, for a number of variants, the functions have been determined.

The HLA-B*07:02 variant is involved in viral clearance and plays an important role in the development of antitumor cells, being associated with tumor regression [33,34,35,36].

The HLA-B*08:01 allele has many immune functions, including viral load regulation, while the HLA-B*13:02 allele is also involved in viral development, having the role of controlling the HIV viral infection [33,34,35,36].

The HLA-B*18:01 allele has the function of controlling antitumor immune response [33,34,35,36].

The HLA-B*27:05 allele plays an important role in long-term protection against viral infection. Also, HLA-B*27:05 in complex with other peptides leads to incapacity of KIR3DL1 to recognize this allele, and the result is increased activation of NK cells during viral infection [37,38,39,40].

Pathological correlations have been described for the HLA-B*15:02 allele, as it is considered a risk factor for Stevens–Johnson syndrome [33,34,35,36].

The HLA-B*27 allele subtypes are associated with ankylosing spondylitis [37,38,39,40]. The HLA-B*27:02 variant is considered a risk factor for ankylosing spondylitis, while HLA-B*27:07 appears to have a protective role against this pathology [37,38,39,40].

The HLA-B*51 allele is associated with an increased susceptibility for developing Behcet’s disease [33,34,35,36].

The HLA-B*57:01 allele is implicated in Abacavir hypersensitivity [33,34,35,36].

These findings and the immunological and pathological correlations need still to be verified for the Romanian population. The implications of the HLA-B*51 allele in the development of Behcet disease are still yet to be fully understood, while the involvement of the HLA-B*18:01 variant in antitumor immune response needs much more research and comprehension and HLA-B*35 alleles have no yet determined correlations.

### 3.3. HLA-C Alleles

The most frequently detected HLA-C alleles in previously analyzed European populations are HLA-C*07 (in most of the countries: England, Albania, Belgium, Croatia, Finland, Germany, Italy, the Netherlands, Norway and the Norway Sami population, Poland, Serbia, Sweden, and Switzerland), while the HLA-C*04 variant is the most common in Greece, Kosovo, and Spain, and the HLA-C*06 (C*06:02) just for the Czech population [14].

In the current research, the HLA-C allele with the highest frequency was HLA-C*07:01:01 (17.36%), but the combined frequencies of all four detected HLA-C*07 variants reach approximately 24% (HLA-C*07:01:01, HLA-C*07:02:01, HLA-C*07:04:01, HLA-C*07:18:01). These results also confirm previous Romanian low-resolution findings stating the HLA-C*07 variant as the main HLA-C allele in Romanians [12,13].

The frequency HLA-C*07:01:01 variant identified in our cohort is one of the highest determined in any previous studies worldwide. If the results of the current research are validated through further studies, this frequency would be the fourth highest globally (17.36%), being surpassed only by the Mexico Hidalgo Mezquital Valley/Otomi population (AF 29%), the USA San Francisco Caucasian population (AF 21%), and the Northern Ireland population (AF 19%). AFs comparable to that detected in our cohort can be observed in populations such as England Blood Donors of Mixed Ethnicity (16.2%), USA Eastern European and Italian Ancestry, Spanish Canary Islands, or Morocco Nador Metalsa populations (all with AFs of approximately 16%), but also in the Polish population (AF approximately 14%) [15].

The HLA-C*04:01:01 allele, the second most common in our cohort (13.47%), has been detected in similar frequencies in populations such as Russian Belgorod Region (13.4%), Polish (12%) and Canary Islands Spanish (15.3%) populations, but also populations from Brasil (13%), India (13%), Kenya (13%), Morocco (12%), South Africa (14%), or Costa Rica (14%) [15].

The HLA-C*12:03:01 (10.69%), as was the HLA-C*07:01:01, was identified in high frequencies in our cohort when compared to other analyzed populations. The detected result would place the frequency observed in the current Romanian study group for the HLA-C*07:01:01 allele as the third highest worldwide, with only the Russian Belgorod region (14.4%) and the China Jingpo Minority (12%) populations revealing greater AFS. The Polish (10.4%) and the Russian Federation Vologda Region (9%) populations also show high frequencies [15].

Even though the HLA-C*04:01:01 is the second most common in our cohort, it is the only HLA-C*04 allele identified, while the HLA-C*06:02 variant, which is the most frequent in the Czech population, also has high frequencies in Romanians as well, being the fourth most observed HLA-C allele (HLA-C*06:02:01—9.44%).

HLA-C alleles have many immune functions, including antiviral immunity and an important role in reproduction.

The HLA-C*01:02 allele is involved in the lysis of infected cells by interacting with KIR receptors (KIR 2DL2 and KIR2DL3) [40,41,42,43,44].

The HLA-C*04:01 allele is associated with antiviral immunity triggering T cell cytotoxic response [40,41,42,43].

The HLA-C*07:02 has an important function in the control of CMV (cytomegalovirus) infection [40,41,42,43].

The HLA-C*06:02 allele is associated with increased susceptibility to developing psoriasis 1 [40,41,42,43].

The implications of HLA-C alleles, in association with KIR receptor variants, in infertility or fertility problems has become a reason for genetic testing in patient couples, with recommendations from geneticists, gynecologists, and fertility of FIV specialists. In this respect, knowing the frequencies of the various HLA-C/KIR constellations for the Romanian population has already become a subject of interest in these medical fields and patient groups. Confirming the results of the current and of previous Romanian studies on greater cohorts would represent an important step towards this goal.

The almost 10% predisposition for psoriasis identified in our cohort through the presence of the HLA-C*06:02:01 allele would single out this HLA-C*06:02 variant for genetic testing in individuals with a diagnosis of a family history of or clinical indications for this disorder.

## 4. Materials and Methods

A total of 420 voluntary donors (Romanians/Caucasians, 61% male, age 43.3 ± 7.7 years old) registered in the National Registry of Voluntary Hematopoietic Stem Cell Donors (RNDVCSH) were included in the study for HLA typing.

Healthy donors who voluntarily registered for stem cell donation in the RNDVCSH between 2020 and 2021 were included in the current research, which was conducted at the Fundeni Clinical Institute in the Medical Analysis Laboratory 2. Written consent from voluntary donors was sought for the processing of evidence and personal data in compliance with the Declaration of Helsinki. The Fundeni Clinical Institute’s Ethics Committee examined and approved this study.

The research team that worked on the project extracted, processed, and statistically examined medical data from each donor’s medical file. Participants in our study were willing donors who did not have any underlying medical conditions. In accordance with the national protocol, we examined each donor’s medical history from their personal medical record. We also examined their biochemical parameters and viral status after donating blood.

The DNA utilized in this study was taken from peripheral blood that was collected in vacutainers containing the anticoagulant EDTA (ethylene-diamino-tetra-acetic acid). The manual DNA extraction method was used to separate DNA from blood. The QIAmp DNA Blood Mini^®^ extraction kit (QIAGEN, Hilden, Germany) was used to extract DNA. This quick and simple method is based on silicon dioxide membranes and allows for the purification of total DNA (genomic, mitochondrial) from bone marrow, cell cultures, leukocyte concentrate, and whole blood.

Each blood sample was thoroughly vortexed, combined with lysis buffer and protease, and then heated to 56 degrees Celsius for 10 min in a thermoblock to promote quick lysis. Following the breakdown of the cell membranes, the DNA was still free in the lysate, with 80% alcohol being added to cause it to precipitate. Since the two materials had different electrical charges, the lysate was placed into tubes that had silicon membranes to which DNA adheres. After adding the elution buffer, which neutralizes the electrical charges, the DNA was purified by several washings and separated from the silicon membrane. Before being used, DNA was separated into tubes and kept at −18 °C. Using an A260 nm/A280 nm ratio between 1.7 and 1.9, which certifies solution purity, and a DNA concentration > 20 ng/µL, an IMPLEN nanophotometer was used to measure the concentration and purity of DNA.

Genotyping of HLA class I alleles (HLA-A, -B, -C) at 6 digits of resolution was performed using the Mia Fora NGS MFlex kit from Immucor.

The MIA FORA NGS MFlex HLA kit (MIA FORATM NGS MFlex) from Immucor (Werfen, France) was used to conduct HLA genotyping using next-generation methods. Sequencing and data analysis, library building, and long-range PCR are the three key steps in this procedure. We amplified the most relevant HLA genes in the long-range PCR step. Adenine nucleotides are added to the ends of each fragment after fragmented probes are used to construct libraries, which improves the ligation of the unique index adapters. To facilitate simple identification during sequencing, every fragment is barcoded. Afterwards, a final amplification of the size-selected library was necessary to guarantee sufficient cluster generation. DNA fragments with 500–900 base pairs were chosen using the Pippin Prep system. Utilizing a Qubit^®^ fluorometer (Thermo Fisher Scientific, Waltham, MA, USA), the concentration was measured prior to final library preparation and adjusted in accordance with the protocol.

A MiniSeq sequencer manufactured by Illumina (San Diego, CA, USA) was used to load the NGS sequencing library once it had been prepared using Illumina reagents. Once sequencing was finished, data were interpreted utilizing the MIA FORA NGS FLEX program (Sirona Genomics, Inc., Mountain View, CA, USA), and two reference databases, Sirona Genomics and IMGT.

## 5. Conclusions

Although the characteristics of HLA class I and II alleles and haplotypes in the Romanian donors are similar to most previously studied European populations, they still retain unique characteristics. Data from this study will be useful in anthropology, immune-mediated diseases, transplantation therapy, and drug hypersensitivity. As the current research was limited to 420 individuals and the MHC region is the most polymorphic of the human genome and can be highly affected by various populational parameters, more extended studies, including larger cohorts, are needed for confirming these findings and expanding them for the entire Romanian population.

## Figures and Tables

**Table 1:** HLA-A genotyping results.

Allele HLA-A	No.	AF (Allele Frequency)
A*02:01:01	188	26.11%
A*01:01:01	90	12.50%
A*24:02:01	84	11.67%
A*03:01:01	70	9.72%
A*11:01:01	45	6.25%
A*32:01:01	45	6.25%
A*26:01:01	33	4.58%
A*68:01:02	21	2.92%
A*23:01:01	18	2.50%
A*25:01:01	17	2.36%
A*29:02:01	16	2.22%
A*33:01:01	12	1.67%
A*33:03:01	12	1.67%
A*30:01:01	10	1.39%
A*31:01:02	10	1.39%
A*29:01:01	8	1.11%
A*02:05:01	6	0.83%
A*02:02:01	4	0.56%
A*66:01:01	4	0.56%
A*68:02:01	4	0.56%
A*30:02:01	4	0.56%
A*02:06:01	3	0.42%
A*02:11:01	3	0.42%
A*24:03:01	3	0.42%
A*02:17:02	2	0.28%
A*03:02:01	2	0.28%
A*30:04:01	2	0.28%
A*02:09:01	1	0.14%
A*24:07:01	1	0.14%
A*34:02:01	1	0.14%
A*02:18:02	1	0.14%

**Table 2:** HLA-B detected alleles.

HLA-B Allele	N	AF
B*18:01:01	81	11.25%
B*51:01:01	78	10.83%
B*08:01:01	56	7.78%
B*35:01:01	36	5%
B*35:03:01	33	4.58%
B*07:02:01	27	3.75%
B*13:02:01	27	3.75%
B*38:01:01	22	3.06%
B*44:02:01	22	3.06%
B*14:02:01	21	2.92%
B*27:05:02	21	2.92%
B*44:03:01	21	2.92%
B*35:02:01	17	2.36%
B*41:01:01	16	2.22%
B*52:01:01	16	2.22%
B*40:02:01	15	2.08%
B*57:01:01	15	2.08%
B*39:01:01	14	1.94%
B*44:05:01	14	1.94%
B*49:01:01	14	1.94%
B*27:02:01	13	1.81%
B*55:01:01	13	1.81%
B*15:01:01	12	1.67%
B*58:01:01	12	1.67%
B*40:01:01	11	1.53%
B*50:01:01	10	1.39%
B*37:01:01	8	1.11%
B*47:01:01	8	1.11%
B*18:05:01	7	0.97%
B*40:06:01	7	0.97%
B*44:27:01	7	0.97%
B*56:01:01	7	0.97%
B*41:02:01	5	0.69%
B*07:05:01	4	0.56%
B*53:01:01	4	0.56%
B*35:08:01	3	0.42%
B*39:06:02	3	0.42%
B*51:07:01	3	0.42%
B*15:03:01	2	0.28%
B*51:05:01	2	0.28%
B*15:17:01	2	0.28%
B*18:03:01	2	0.28%
B*45:01:01	2	0.28%
B*54:01:01	2	0.28%
B*46:01:01	1	0.14%
B*48:01:01	1	0.14%
B*18:04:01	1	0.14%
B*39:05:01	1	0.14%
B*58:02:01	1	0.14%

**Table 3:** HLA-C alleles identified through the current study.

HLA-C Allele	N	AF
C*07:01:01	125	17.36%
C*04:01:01	97	13.47%
C*12:03:01	77	10.69%
C*06:02:01	68	9.44%
C*02:02:02	63	8.75%
C*01:02:01	39	5.41%
C*07:02:01	37	5.13%
C*15:02:01	36	5%
C*03:04:01	20	2.77%
C*08:02:01	20	2.77%
C*14:02:01	20	2.77%
C*03:03:01	19	2.63%
C*12:02:02	18	2.50%
C*05:01:01	12	1.66%
C*16:01:01	11	1.52%
C*03:02:01	10	1.38%
C*07:04:01	10	1.38%
C*17:01:01	7	0.97%
C*16:07:01	6	0.83%
C*17:03:01	5	0.69%
C*15:05:02	4	0.55%
C*15:13:01	4	0.55%
C*16:02:01	4	0.55%
C*15:04:01	3	0.41%
C*12:12	1	0.13%
C*07:18:01	1	0.13%
C*15:72	1	0.13%
C*08:03:01	1	0.13%
C*14:03:01	1	0.13%

## Data Availability

Restrictions apply to the datasets for the moments. The datasets presented in this article are not readily available because the data are part of an ongoing study. Requests to access the datasets should be directed to andreea.m.caragea@gmail.com.
